# 

**Published:** 1895-03-30

**Authors:** 


CONTENTS.

**Is Insanity Increasing in Scotland?** - 451
**The Dangers of Electricity** - 452
**Origin and Classification of Aneurisms.** By A. PEARCE GOULD, F.R.C.S. - 453
**The Medical Care of Idiots, Imbeciles, and Feeble-Minded Children.** By G. E. SHUTTLEWORTH, B.A., M.D., &c. 454
**Medical Progress and Hospital Clinics:**
- The Plague of Consumption - 457
- The Treatment of Chronic Cystitis - 458
- PROGRESS IN MEDICINE.—Diseases of the Circulatory System - 459
- PROGRESS IN SURGERY.—Genito-Urinary Surgery - 460
- NEW APPLIANCES AND THINGS MEDICAL - 462

**Annotations.**—Should First Cousins Marry?—The Religious Question at the Tottenham Hospital—Spring Headaches - 455
**Notes and News** - 456
**The Institutional Workshop:**
- HOSPITAL ADMINISTRATION.—Pay Wards in General Hospitals - 463
- THE STORY OF THE INSANE FROM YEAR TO YEAR - 464
- PRACTICAL DEPARTMENTS.—Portable Fire Escape - 465
- HOSPITAL FESTIVALS AND MEETINGS - 465
- CONSTRUCTION NOTES - 466

**Scraps and Gleanings** - 466

**The Book World of Medicine and Science** - 466

**The Student of Medicine** - 467

EXTRA SUPPLEMENT.—THE NURSING MIRROR.

**News from the Nursing World.**—Under Royal Patronage—Hospital for Consumption—King's College Hospital—Divided Duties—St. Catherine's Home—Prompt Measures at Hinckley—A Matron's Opinion of Presentations—The "Nurses' Journal"—Caged Orphans—Indian Lady Doctors—Short Items - clxxxix

**Death in Our Ranks** - cxcii

**Elementary Anatomy and Surgery for Nurses.** By W. MCADAM ECCLES, M.B., M.S., F.R.C.S. - cxci

**Where to Go** - cxcii

**Notes from Holland** - cxcii

**A Great Movement: The Nurses Co-operation** - cxciii

**The Metropolitan Hospital** - cxciii

**Everybody's Opinion—Appointments** - cxciv

